# Probiotic, Postbiotic, and Paraprobiotic Effects of *Lactobacillus rhamnosus* as a Modulator of Obesity-Associated Factors

**DOI:** 10.3390/foods13223529

**Published:** 2024-11-05

**Authors:** Gabriela López-Almada, María Esther Mejía-León, Norma Julieta Salazar-López

**Affiliations:** Facultad de Medicina de Mexicali, Universidad Autónoma de Baja California, Dr. Humberto Torres Sanginés, Centro Cívico, Mexicali 21000, BCN, Mexico

**Keywords:** supernatants, *Lactobacillus rhamnosus* GG supernatant, oxidative stress, adiposity, inflammation, adipokines

## Abstract

Obesity is a pandemic currently affecting the world’s population that decreases the quality of life and promotes the development of chronic non-communicable diseases. *Lactobacillus rhamnosus* is recognized for multiple positive effects on obesity and overall health. In fact, such effects may occur even when the microorganisms do not remain alive (paraprobiotic effects). This raises the need to elucidate the mechanisms by which obesity-associated factors can be modulated. This narrative review explores recent findings on the effects of *L. rhamnosus*, particularly, its postbiotic and paraprobiotic effects, on the modulation of adiposity, weight gain, oxidative stress, inflammation, adipokines, satiety, and maintenance of intestinal integrity, with the aim of providing a better understanding of its mechanisms of action in order to contribute to streamlining its clinical and therapeutic applications. The literature shows that *L. rhamnosus* can modulate obesity-associated factors when analyzed in vitro and in vivo. Moreover, its postbiotic and paraprobiotic effects may be comparable to the more studied probiotic actions. Some mechanisms involve regulation of gene expression, intracellular signaling, and enteroendocrine communication, among others. We conclude that the evidence is promising, although there are still multiple knowledge gaps that require further study in order to fully utilize *L. rhamnosus* to improve human health.

## 1. Introduction

Obesity is a pandemic that is currently affecting the world’s population. It is the basis for the development of chronic non-communicable diseases (NCDs) like diabetes and cardiovascular and metabolic diseases, among others, in addition to psychological and mobility problems, which considerably reduces the quality of life of those who suffer from it [1]. It is, therefore, necessary to identify alternatives that can counter obesity from different fronts, in particular, by searching for molecules capable of exerting beneficial anti-obesogenic effects, which have the potential to become serious alternatives for the prevention and treatment of obesity.

Probiotics can be such alternatives against obesity, since they have shown anti-obesogenic effects, in addition to also mitigating some of its associated comorbidities like diabetes, dyslipidemia, colorectal cancer, metabolic syndrome, and others [2]. *Lactobacillus rhamnosus* GG (LGG) is one of the most well-studied probiotics with multiple reported anti-obesity-related actions. Some of the particular mechanisms of action by which these effects are exerted, including modulation of oxidative stress (OS) [3,4], inflammation [5], intestinal dysbiosis [6], insulin resistance [7], adipokines like adiponectin [8] and leptin [9], weight gain in vivo [10], and in humans [11], all of them metabolic derangements that are associated with obesity. This creates an opportunity to establish preventive and therapeutic strategies in obesity care.

In addition to these health effects, a new field of research has developed from probiotic derivatives in recent years, in which the origin, composition, mechanism of action, and potential benefits of these derivatives are evaluated. This has allowed for the rise of internationally defined terms like “postbiotics” and “paraprobiotics” [12]. Postbiotics are defined as inactivated microbial cells and/or their components, with or without metabolites, that confer health benefits [12]. The term “paraprobiotic” has also been used to define an inactivated microorganism that is non-viable, either ruptured or complete, and also confers health benefits [13,14]. With this consideration, in this review, the term “postbiotic” will refer to secreted metabolites like cell-free supernatants, cell-free extracts, enzymes, peptides and proteins, and short-chain fatty acids (SCFA), among others; “paraprobiotic” will refer to the inactivated form of the bacteria, such as heat-killed, or to cell structures like peptidoglycans, cell walls, or cell lysates.

Continuing research on these topics has been accompanied by particular questions: are probiotics, postbiotics, or paraprobiotics the inducers of benefits? Do the benefits originate from a dual mechanism between the microorganism and its postbiotics or paraprobiotics, or is it due to the sum of both? Or, to what extent is the effect of the postbiotics or paraprobiotics limited? The above indicates that the boundary between the role of *L. rhamnosus* and its derivatives on health is not clear.

Analyzing the effects of probiotics and their derivatives on the main processes involved in obesity may allow the identification of fields of action that could contribute to understanding their impact on the obese state, as well as on the development and progression of associated pathologies. Some of the molecular and cellular processes that are known to be altered in these associated pathologies are OS and inflammation. They usually occur together and promote each other, which establishes a key vicious cycle in the development and progression of the aforementioned diseases. The present narrative review summarizes recent findings regarding the effect of the probiotic *L. rhamnosus* GG. Its postbiotic effect on the modulation of adiposity, weight gain, OS, inflammation, regulation of adipokines, satiety, and maintenance of intestinal integrity are specifically addressed to provide a better understanding of the mechanisms of action and contribute to streamlining its clinical and therapeutic application.

## 2. Adiposity

Obesity is characterized by excessive adiposity. Its distribution is also relevant in predicting health risks; central adiposity is associated with cardiovascular and metabolic risks [15]. Adiposity is associated with regular over-consumption of energy-dense diets, especially those whose energy derives mainly from saturated fats, long-chain fatty acids, and refined simple carbohydrates; these promote the storage of surplus energy as triglycerides in white adipose tissue adipocytes. The excessive accumulation of triglycerides, coupled with sustained high dietary energy intake, contributes to hypertrophy of the adipose tissue. Hypertrophied adipose tissue is associated with hypoxia, which results in dysfunction of this tissue, thereby promoting the development of OS and inflammation, which are the basis for the development of chronic NCDs [16,17]. Therefore, reducing triglyceride storage in adipocytes by regulating adipogenic and thermogenic processes could be part of a reasonable therapeutic approach to counteract obesity.

Oral administration of *L. rhamnosus* probiotics results in anti-obesogenic effects. In this regard, the administration of *L. rhamnosus* CGMCC1.3724 (2 capsules/day, 1.6 × 10^8^ CFU/capsule) reduced body weight in obese women, an effect that was associated with a reduction in adipose mass and circulating leptin concentrations, in addition to the high relative abundance of the family *Lachnospiraceae* in feces [11]. Additionally, *L. rhamnosus* HA-114 administered for 12 weeks reduced plasma values of insulin, HOMA-IR, LDL- cholesterol, and triglycerides and modulated eating behaviors (binge eating and food cravings), but it did not induce a reduction in body weight or fat mass in adults. However, the long-term behavioral and metabolic changes could directly influence weight control and adiposity reduction [18].

Adipogenesis involves a process in which preadipocytes become mature adipocytes (differentiation) that are capable of accumulating intracellular lipids through the regulation of transcription factors. Some of the key transcription factors involved in this process are the nuclear hormone receptor peroxisome proliferator-activated receptor γ (PPARγ) and CCAAT/enhancer binding proteins (C/EBPα), whose inactivation limits adipogenesis [19]. PPARγ also enhances the expression of mature adipocyte marker genes like ap2 [20].

AMP-activated protein kinase (AMPK) regulates cellular energy. Upon phosphorylation, it inhibits fatty acid synthesis by inactivating acetyl-CoA carboxylase (ACC-1) and de novo lipogenesis, which includes the inactivation of fatty acid synthase (FAS). Otherwise, AMPK promotes FA oxidation [21].

With in vivo models (mice), *Lactobacillus* probiotics have been shown to reduce subcutaneous and mesenteric adipose tissue weight and dyslipidemia, as well as prevent negative changes on the adipokine profile, even when the animals were exposed to an obesogenic diet [22]. The mechanism behind these effects lies in down-regulating white adipose tissue protein expression of PPAR-γ, aP2, C/EBP-α, p-AMPK, p-ACC, and FAS [22]. This mechanism is also apparent from *L. rhamnosus* probiotic fermented products in vitro [23]. In addition to these probiotic results, postbiotic forms (*L. rhamnosus* DS0508 supernatant) increase the expression of brown adipose tissue marker proteins involved in thermogenesis and browning process, both in vitro and in vivo, such as uncoupling protein 1 (UCP-1) and PGC1α [24] through phosphorylation of participating proteins. On brown adipose tissue, UCP-1 promotes thermogenesis and fatty acid oxidation. PPAR-γ coactivator 1α (PGC-1α) is a transcription factor that coordinates the expression of other thermogenic genes, such as cAMP response element-binding protein (CREB) and UCP-1. Additionally, protein kinase A (PKA) stimulates the phosphorylation of CREB and activates other thermogenic transcription factors that induce UCP-1 transcription [25].

Regarding the paraprobiotic form (HK *L. rhamnosus* 86), it has also been shown to reduce lipid accumulation and triglyceride content in vitro, according to a mechanism related to changes exerted at the transcriptional level (mRNA down-regulation) of PPAR-γ, C/EBPα, and aP2 [26], which altogether inhibit adipogenesis, adipocyte-differentiation, and lipid-synthesis pathways.

The LGG exopolysaccharide is able to reduce intracellular triglyceride accumulation in the 3T3-L1 cell line by up to 50% (vs CT, *p* < 0.05), associated with a down-regulation of lipogenic gene expression (PPAR-γ, ap2, FAS, DGAT1), which is maintained with in vivo assays [27]. The adipogenic down-regulation is TLR2-dependent and is associated with an increase in TLR2 gene expression, suggesting an EPS-TLR2-signaling regulatory mechanism, although no significant changes in lipolysis were observed. This study further investigated if other components of the exopolysaccharide, rather than just its monosaccharides, are responsible for this effect.

Extracellular vesicles (EVs) derived and isolated from *L. rhamnosus* supernatants have been shown to contain proteins, enzymes, and lipoteichoic acid [28]. Lipoteichoic acid (10 μg/mL) isolated and purified from other strains (*Bifidobacterium animalis* subsp. lactis CECT 8145 BPL1) induced a significant decrease in fat deposition (26.6%, *p* < 0.001) and triglyceride content (*p* < 0.01) in the nematode *C. elegans* through a mechanism involving the insulin/IGF-1 pathway [29]. Accordingly, the protective effects of postbiotics or the paraprobiotic form could be associated with the presence of bacterial cell wall components, such as lipoteichoic acid and polysaccharides [30], which could modulate inflammation. Therefore, the anti-adipogenic and anti-inflammatory effects of lipoteichoic acid derived from *Lactobacillus* spp. strains merit further evaluation for their postbiotic application against obesity.

Taken together, it appears that all these forms share a translational–transcriptional-regulatory mechanism of action on adipogenic and thermogenic pathways, resulting in a similar antiadipogenic effect in vitro and in vivo. Yet, the paraprobiotic and postbiotic forms provide a potential alternative to live bacteria [31] and may offer potential for significant therapeutic application [32].

Metabolomic analyses have revealed the presence of other compounds produced by *L. rhamnosus*, in addition to lipoteichoic acid. These include inosine, pyroglutamic acid, and SCFA, among others [33,34]. Table 1 summarizes *L. rhamnosus*-based treatments, the main compound(s) reported, and various effects relevant to the modulation of obesity.

## 3. Adipokines

The dysregulation of adipose tissue in the obese state is accompanied by changes in the serum concentration of adipokines, mainly adiponectin and leptin, which can contribute to the progression of metabolic disorders. Obesity is known to be accompanied by decreased serum adiponectin and increased serum leptin. Leptin is secreted in proportion to the mass of white adipose tissue and plays multiple physiological roles, including in the hunger–satiety pathway by exerting a central anorexigenic effect [66]. Increased leptin in obesity is also associated with leptin resistance in the central nervous system, which decreases its anorexigenic effects despite its high peripheral concentration [67]. The mechanisms behind this resistance include a decreased serum leptin transport across the blood–brain barrier that limits the effect of leptin on satiety-regulatory structures in the hypothalamus, a down-regulation of the leptin-signaling pathway through the LepRb leptin receptor (a JAK2–STAT3 pathway), hypothalamic inflammation, and endoplasmic reticulum stress [67]. Particularly, down-regulation of the leptin-signaling pathway further limits the transcription of the suppressor of cytokine signaling 3 (SOCS3), resulting in a diminished feedback mechanism and signaling, accompanied by decreased transcription of the anorexigenic neuropeptide gene POMC, all of which contribute to the satiety dysregulation [67]. Hyperleptinemia is also characterized by having a proinflammatory effect by stimulating the secretion of cytokines like TNF-α and IL-6 [68]. In contrast to leptin, adiponectin tends to be decreased in obesity, and, since one of its main actions is to regulate glucose metabolism, decreased adiponectin tends to promote a diabetic effect. Serum levels of both adipokines have been used as biomarkers of obesity and metabolic syndrome [66].

LGG probiotics are able to increase serum leptin and induce its expression in the intestinal epithelium [69]. In a mice model that fed an HFD for 12 weeks, *L. rhamnosus* (BST-L.601) is capable of decreasing serum leptin in its probiotic and probiotic fermented product to a similar extent (27.8% vs. 24%, respectively) [23]. Likewise, LGG has been shown to be able to restore the response to exogenous leptin in an in vivo model of HFD-induced leptin resistance [9], suggesting that LGG supplementation may reduce leptin resistance, commonly observed in obesity.

Regarding adiponectin, probiotic LGG supplementation prevented a decrease in adiponectin mRNA expression in adipose tissue and decreased serum protein levels induced by a high-fat diet in vivo [7]. A modulating effect was also observed when *L*. *rhamnosus* MTCC 1423 (10⁹ CFU/day) was administered in combination with a Kyolic-aged garlic extract (200 mg/kg orally for 5 weeks) to Wistar rats as a synbiotic, increasing adiponectin mRNA expression in adipose tissue while significantly reducing (*p* < 0.0001) mRNA expression of proinflammatory molecules like TNF-α, leptin, resistin, iNOS, and TLR4 [70].

Regarding postbiotics, an in vivo model (C57BL/6J mice) of metabolic syndrome, the supplementation with LGG supernatant in the diet (dose equivalent to 10⁹ CFU/day for 15 weeks) increase in serum adiponectin (up to 4.2-fold, (*p* ˂ 0.001) [71], such as a decrease in body weight gain (*p* ˂ 0.05) and lean mass. The changes in serum adiponectin were associated with increased serum mRNA and protein of hepatocyte-derived fibroblast growth factor 21 (FGF21) by up to 3.2-fold (*p* ˂ 0.01). This aligns with the findings on the supplementation of probiotic LGG and paraprobiotic (HK) in a rat model with an obesogenic diet, which prevented decreased adiponectin serum levels and decreased serum FGF-21 [72]. In this regard, the administration of FGF21 analogues has induced adiponectin gene expression in mice, which stimulates the secretion of this adipokine from adipocytes through a PPAR-γ-dependent mechanism [73]. Furthermore, the modulatory effects of probiotic supplementation (LGG) on serum adiponectin levels were abolished in FGF21-deficient (KO) mice [8]. This suggests that adiponectin up-regulation is considerably mediated by this metabolic regulator. Thus, a dysfunction in the adiponectin–FGF21 axis has been proposed in the pathogenesis of MetS and obesity [74]. LGG supernatant could induce FGF21 expression in the liver, which subsequently increases adiponectin expression in adipose tissue. However, other signaling pathways should be considered [71].

In a similar study with the same in vivo model, LGG supernatant was able to restore cardiac gene expression of adiponectin receptor AdipoR1 (*p* ˂ 0.05), while no changes were observed in AdipoR2 [75]. The authors of this study propose that the observed effect is indirectly mediated by increased serum adiponectin.

In addition to an up-regulation of adiponectin secondary to changes in adipose tissue mass and weight, no other studies have determined alternative mechanisms by which LGG supernatant could directly regulate adiponectin mRNA or adiponectin serum levels. However, molecules such as polyphenols and alkaloids are capable of up-regulating adiponectin mRNA expression through an Akt/FOXO1 and AMPK pathway [76,77]. However, these mechanisms remain to be confirmed. Also, the down-regulation of adiponectin transcription by proinflammatory cytokines could be abolished, secondary to the anti-inflammatory effects of postbiotics [78].

## 4. Oxidative Stress (OS)

Several studies have shown an association between obesity and an OS state [79]. OS results from an imbalance between the antioxidant system and the generation of free radicals or reactive species (RS). RS is essential for redox homeostasis and vital physiological processes like cellular proliferation and differentiation. The main source of RS in cells is mitochondria, although some are derived from signal transduction or other cellular defense processes. Free fatty acids and hyperglycemia promote an increase in β-oxidation, resulting in an excessive flow of electrons that contribute to the formation and accumulation of RS, leading to a redox imbalance in the cellular environment [79].

OS and mitochondrial dysfunction contribute to endoplasmic reticulum dysfunction, which, initially through the activation of the unfolded protein response (UPR) and, particularly, through protein kinase PERK, detects RS and attempts to attenuate the state of cellular stress. However, in the face of persistent negative stimuli, UPR eventually becomes insufficient to maintain cellular homeostasis, which allows the development of endoplasmic reticulum stress, apoptosis, or necrotic cell death [80].

Excess RS can cause cellular damage since it is able to interact with biomolecules and structures like cell membranes, proteins, and DNA. However, the body has an endogenous antioxidant system that prevents the development of this imbalance, which includes enzymes like superoxide dismutase (SOD), catalase (CAT), and glutathione peroxidase (GPx), in addition to molecules like glutathione (reduced-form GSH; oxidized-form GSSG). To activate the synthesis and production of this antioxidant system, RS regulates gene expression through nuclear factor erythroid 2 (Nfr2) and nuclear factor κB (NF-κB) [81]. In addition to the endogenous antioxidant system, some exogenous food-derived molecules possess antioxidant capacity, such as vitamins A, C, and E, as well as phenolic compounds and other phytochemicals [82].

Certain probiotics have demonstrated an antioxidant effect [83,84]. For example, *L. rhamnosus*, *L. paracasei*, and *L. fermentum* demonstrated antioxidant capacity, as well as survival in an induced OS state (exerted by exposure to hydrogen peroxide). Although the mechanism of antioxidant capacity in these strains was not specified, the set of these results indicates that the antioxidant properties are strain-specific [85,86].

*L. paracasei* M11-4, in particular, has shown antioxidant mechanisms by genetic up-regulation of thioredoxin and glutathione systems, as well as antioxidant enzymes in vitro and in vivo [87]. *L. fermentum* CQPC04 in fermented soy milk up-regulated mRNA expression of Nrf2, heme oxygenase-1, CAT, and SOD in vivo [88], while another strain increased the activity of antioxidant enzymes and decreased MDA [89].

LGG, provided by oral gavage to mice for 2 weeks, revealed an increase in Nrf2 immunoreactivity in the liver, which confirms its translocation and activation [90], with Nrf2 mRNA up-regulation later being confirmed by another in vivo study [91]. In both studies, LGG exerted a remarkable protective effect against acetaminophen-induced hepatotoxicity. Additionally, supplementation with LGG or LGG supernatants on a high-fat and fructose diet in an in vivo model exerted a similar protective effect against the accumulation of OS biomarkers (4-HNE and 3-NT) induced by intermittent hypoxia and was associated with the preservation of Nrf2 and downstream gene expression, suggesting that LGG and LGG supernatant are Nrf2 activators, which could contribute to an antioxidant response [75].

On the other hand, LGG postbiotic forms (postbiotics obtained by ultrasonic treatment and a supernatant) showed antioxidant capacity. However, supernatants showed higher antioxidant capacity compared to the postbiotics obtained by ultrasonic (*p* < 0.05), as evidenced by reducing activity (168.0 ± 2.6 vs. 71.9 ± 1.2 mmol cysteine equivalents), DPPH scavenging activity (77.7 ± 5.3 vs. 26.6 ± 0.2%), OH (64.0 ± 2.9 vs. 33.8 ± 1.4%) and O_2_ (22.6 ± 0.4 vs. 18.9 ± 0.1%), as well as lipid peroxidation-inhibition capacity (22.3 ± 1.2 vs. 14.3 ± 0.4%) (*p* < 0.05) [92]. Overall, a higher reducing activity of the supernatant and a higher superoxide anion scavenging activity by postbiotic obtained by ultrasonic treatment were observed [92]. LGG supernatant (6 × 10⁹ CFU) was able to reduce ER production and prevent changes in the GSSG:GSH ratio of total glutathione in rotavirus (RV)-infected Caco-2 cell lines when compared to the virus-only group (*p* < 0.05), which was similar to that observed with LGG probiotic treatment [93]. A total prevention of RV-induced chloride ion secretion by the supernatant was achieved when compared to LGG (*p* < 0.05) [93].

In addition, in vitro LGG supernatant prevented the production of RS similar to N-acetylcysteine (*p* < 0.0005) and the decrease of GSH levels (*p* < 0.0005) and lipid peroxidation (*p* < 0.0005) [94]. Altogether, these results indicate the inhibitory effect of RS and OS induced by the probiotic and postbiotics of *L. rhamnosus*.

Regarding postbiotics contained in the supernatant, coffee extract prepared and fermented with LGG contains molecules like pyroglutamic acid and ferulic acid [33]. Pyroglutamic acid has been reported to be a precursor for the biosynthesis of GSH [95], while ferulic acid is an antioxidant capable of reducing OS and scavenging synthetic free radicals (DPPH, ABTS) and those of biological interest (peroxyl), as well as modulating enzymes of the endogenous antioxidant system and increasing the antioxidant potential of plasma in HFD-fed rats [54,96,97]. Ferulic acid reduced methotrexate (MTX)-induced hepatic OS in a murine model by decreasing reactive oxygen species, MDA, and NO. It also displayed anti-inflammatory effects by lowering serum levels of TNF-α and IL-1B and liver NF-κB p65, Bax, and caspase-3, as well as increased Blc2, Nrf2, NQO1, HO-1, and PPARγ [98].

Other postbiotics produced by *L. rhamnosus* with the capacity to modulate OS are vanillic acid, 4-vinylphenol, and inosine. Vanillic acid, in particular, is an antioxidant with the ability to eliminate free radicals, activate the Nrf2 pathway, and inhibit the inflammatory HMGB1/NF-κB axis [99]. Meanwhile, 4-vinylphenol stabilizes the DPPH radical [100] and inosine can increase plasma antioxidant capacity in patients with early Parkinson’s disease [101].

## 5. Inflammation

In obesity, OS contributes to the inflammatory process by phosphorylating IkBα, the main inhibitor of NF-κB, allowing its translocation to the nucleus with subsequent activation of pro-inflammatory gene-transcription factors [16]. Inflammation initially aims to provide an adaptive response after cellular damage, perceived by loss of cellular integrity or other signals. However, persistent, low-grade chronic inflammation has been shown to be a constant variable in obesity and associated diseases, such as metabolic dysfunction-associated fatty liver disease (MAFLD), insulin resistance, cancer, and T2DM [102].

Hypertrophied and dysfunctional adipose tissue in the obese state is characterized by infiltration of macrophages and adipocytes capable of secreting proinflammatory cytokines such as TNF-α, IL-1, and IL-6 [103]. TNF-α mainly derives from macrophages and activates the NF–кB pathway to modulate the production of inflammatory mediators [104]. In addition, monocyte chemoattractant protein type 1 (MCP-1), an initiator of monocyte chemotaxis and migration, amplifies all these responses. Associated with these, TNF-α and IL-6 derived from adipose tissue inhibit insulin signaling, which promotes the development of T2DM secondary to obesity [105].

Oral administration of LGG in its probiotic form has shown inflammation-modulating effects on cytokines and genes involved in obesity-related inflammation. With in vivo models of metabolic disorders, LGG regulates tissue gene expression of inflammatory cytokines, such as NF-κB-p65, iNOS, TNF-α, IL-1β, and COX-2 [106,107]. In human studies, LGG decreases serum inflammatory cytokines (IL-6, TNF-α, and IL-1B) [108,109,110], which appears to be secondary to the regulation of dysbiosis, gut permeability, and reduction of chronic inflammation [109]. This could also be secondary to the regulation of gene expression in specific tissues. However, tissue gene expression studies in humans are limited.

Other inflammatory pathways seem to be modulated by *L. rhamnosus* probiotics in vitro. *L. rhamnosus* MG4644 anti-inflammatory effects (reduced mRNA expression of IL-4, IL-5, IL-25, and IL-33) on TNF-α/IFN-γ-induced HaCaT keratinocytes were attributed to a decreased phosphorylation of NF-κB and IκB, as well as the diminished phosphorylation of ERK and p38 of the MAPK pathway [111].

On the other hand, a paraprobiotic (HK) of *L. rhamnosus* (Lr-0601) significantly reduced serum levels of proinflammatory cytokines (IL1-β, TNF-α, IL-6, and IL-8), coupled with a decrease in colonic mRNA expression in an in vivo ulcerative colitis model [112]. These effects were similar to those observed from the probiotic form [113].

Besides reducing proinflammatory cytokine levels and expression, postbiotic and paraprobiotic forms are also able to promote the production of anti-inflammatory cytokines and prevent their downregulation, which seems to be a common activity of other *Lactobacillus* strains. In this regard, probiotic *L rhamnosus* PL60 increased IL-10 levels, while decreasing TNF-α production in HT-29 cells treated with LPS. An in vivo evaluation with LPS-treated mice showed a significant increase in IL-10 serum concentrations, as well as a significant decrease in serum TNF-α concentrations [113]. Particularly, LGG postbiotics (secreted proteins) and paraprobiotics (HK bacterial cells) were capable of modulating inflammation via the production of IL-10 in an in vitro model [114]. However, IL-10 levels induced by the secreted proteins were superior to those obtained with the paraprobiotic over the same period, suggesting a greater ability of the postbiotic to modulate the synthesis and release of this cytokine. This could be explained since secretory proteins may directly interact with mucosal cells and modulate signaling pathways that protect the intestine, further modulating inflammation [114].

Other inflammatory pathways could be implicated in this regulation. *L rhamnosus* GR-1 supernatant in a human placental trophoblasts cell model treated with LPS increased IL-10 levels associated with an up-regulation of STAT3 and p38 phosphorylation. On the contrary, JAK and p38 inhibitors abolished IL-10 concentration [115]. IL-10 up-regulation was not associated with the inhibition of RTK–MAPK and NF–kB pathways, which suggests that *L. rhamnosus* supernatant-associated IL-10 up-regulation is mediated by the JAK/STAT pathway to further regulate gene transcription of this cytokine.

An *L. rhamnosus* MG4511 supernatant used on UVB-exposed skin fibroblast Hs68 cells diminished the mRNA expressions of IL-6 and IL-1β, partially by suppressing OS and by inhibiting MAPK signaling by downregulation of the protein expressions of p-ERK, p-JNK, and p-p38 [116].

Taken together, these results suggest that the systemic modulation of proinflammatory cytokines (e.g., in serum) by different probiotic, postbiotic, and paraprobiotics of *L. rhamnosus* are mediated by downregulation at the gene level, partially through the TLR4/NF–κB pathway, which is a central regulator of inflammatory gene expression in metabolic disorders, including obesity [117]. Other inflammatory pathways like the ERK/JNK/p38 pathway may also play a role, while the up-regulation of anti-inflammatory cytokines may result from other regulatory pathways.

Postbiotic forms have also demonstrated an anti-inflammatory effect in models of acute and subacute inflammation. This is particularly relevant since targeting chronic inflammation associated with obesity could be a therapeutic strategy. Postbiotics (supernatant) derived from LGG and *L. reuteri* have been shown to suppress the release of proinflammatory IL-1β by 53–60% in an in vitro model of RAW-ASC macrophages (transfected with apoptosis-associated spike-like protein) [118]. Since this type of macrophage involves the activation of the NLRP3 inflammasome [118], which is characteristic of the chronic low-grade systemic inflammation present in obesity and insulin resistance, the study of postbiotics as a therapeutic option in obesity deserves further investigation [119]. On the other hand, in a preventive acute inflammatory model of carrageenan-induced rat paw edema [120], a previous oral administration of postbiotics of *L. rhamnosus* and *L. plantarum* showed a slight decrease in paw thickness and edema, inferior to those observed by the probiotic form [120]. However, histological inflammatory changes confirmed the anti-inflammatory effect of postbiotics. This suggests that postbiotics may locally modulate inflammatory mediators or autacoids as well. In support of this, LGG is able to regulate the arachidonic acid pathway, including the leukotriene pathway and COX-2 expression and prostaglandin E2 secretion in the Caco-2 cell line, likely through TLR-MyD88 signaling [121]. These findings could partly explain the analgesic response in the findings of *L. rhamnosus* PB01 probiotic supplementation by oral gavage in diet-induced obese female mice for 4 weeks, which significantly regulated pressure-pain thresholds with reduced pain sensitivity [122].

It is noteworthy to note that the peptidoglycan of the *L. rhamnosus* CRL1505 strain normalized serum levels of TNF-α, IL1-β, IL-6, and IL-10 in an in vivo mouse model, when compared to the non-viable form (exposed to ultraviolet radiation) and the intact cell wall. These findings suggests peptidoglycan as the main cellular component associated with the systemic immunomodulatory effects. However, the same research team later reported that peptidoglycan-mediated effects were not observed in other strains like *L. rhamnosus* CRL534 or *L. plantarum* CRL1506 [123], suggesting that these benefits are strain-specific.

As a possible disadvantage, paraprobiotic forms of *L. rhamnosus* may induce proinflammatory effects. *L. rhamnosus* Lr-32 paraprobiotic increases the transcription of genes like TNF-α, IL-1β, and IL-6 in gingival epithelial cells (OBA-9) [124]. This response could be due to the presence of cellular components in the paraprobiotic, including proteins, extracellular polysaccharides, peptidoglycans, and other cell wall components [125], which may have the ability to activate TLRs through pattern-recognition receptors (PRRs) and stimulate an immune response [126].

On the contrary, other paraprobiotics, such as LGG exopolysaccharide, showed no cytotoxic activity in an in vitro study and showed a protective effect against oxidative stress by increasing survival rates [127]. In support of this, in another study, LGG exopolysaccharide did not change the expression of inflammatory genes like TNF-α, MCP-1, and IL-6 in 3T3L-1 adipocytes and in white adipose tissue in mice [27]. Postbiotic forms such as supernatants contain metabolized or secreted products or enzymes that may lack this antigenicity and response [124], as well as hydrogen peroxide, bacteriocins, and bioactive compounds that may further restrict virulence or pathogenicity of other pathogenic microorganisms [125].

**Figure 1:**
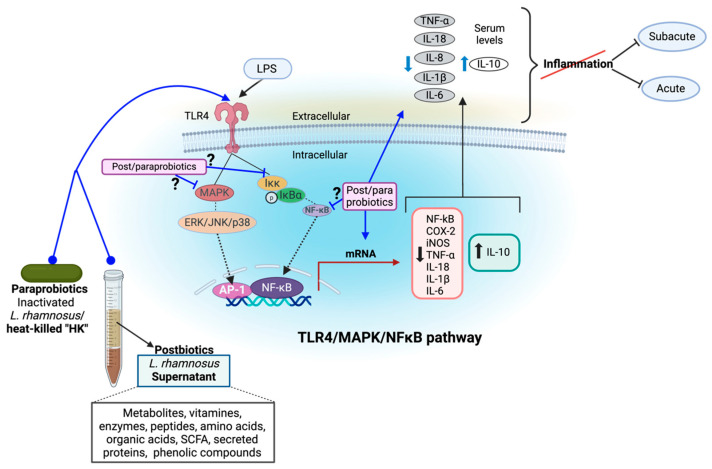
Potential anti-inflammatory mechanisms of postbiotics and paraprobiotics derived from *L. rhamnosus*. *L. rhamnosus* postbiotics or paraprobiotic forms have demonstrated the ability to modulate inflammatory pathways, including TLR4/MAPK/NF-κB, which is associated with the presence of LPS. This is done by modulating the mRNA of various tissues, such as adipose tissue and liver, which explains the changes in serum levels of the interleukins involved. The evidence derives from acute and subacute inflammation models. However, the precise mechanism of modulation at upstream regulatory points of this pathway remains to be elucidated. “?” refers to post- and paraprobiotics mechanisms that still need further research. Custom image created with BioRender.

Figure 1 summarizes the potential inflammatory mechanisms of postbiotics and paraprobiotics derived from *L. rhamnosus* reported in the literature.

## 6. Gut Environment Disturbance

The intestinal epithelium is a semipermeable barrier that, under normal conditions, selectively regulates the entry of molecules into circulation. However, some factors like Westernized diets, stress, or the use of antibiotics can alter the composition of the microbiota and, thus, intestinal homeostasis [128]. It has been proposed that high-fat diets modify the composition and function of the intestinal microbiota, which can induce metabolic deregulation and inflammation that may contribute to the development of obesity and associated chronic non-communicable diseases [129]. Among the microbiota changes associated with obesity, a decrease in diversity and alterations in the *Firmicutes–Bacteroidetes* ratio have been described in both mice and humans. At the genus level, an increase in the relative abundance of microorganisms like *Akkermansia*, *Faecalibacterium*, *Oscillibacter*, and *Alistipes*, as well as a decrease in some *Lactobacillus* species, have also been identified [130]. In these situations, imbalanced components of the microbiota and their products can increase paracellular permeability by promoting thinning of the mucus layer and the breakdown of tight junctions. For example, there is a decreased production of beneficial SCFA, such as butyrate, in the dysbiotic state. This metabolite contributes to the maintenance of barrier integrity by positively modulating the expression of junction proteins like claudin-1, claudin 7, and zonula occludens-1 and -2 (ZO-1, ZO-2). In the obese state, butyrate-producing intestinal bacteria decrease in proportion, leading to changes in the colonic fermentation pattern and, consequently, to a state of intestinal hyperpermeability known as leaky gut syndrome [131]. Other microbiota metabolites, such as indole and its derivatives, are decreased in obesity, particularly as a result of the reduction of commensal bacteria like *Lactobacillus* spp. Compounds like indole-3-lactic acid (ILA), indole-3-aldehyde (I3A), and indole-3-acetic acid (IAA) regulate the expression of anti-inflammatory genes by acting as ligands of aryl hydrocarbon receptors (AhR), which act as transcription factors. Consequently, in obesity, the protective function of microbiota products is compromised, thereby enhancing the proinflammatory state [132].

In addition to altered permeability and dysbiosis, the disturbance of the gut environment related to leaky gut syndrome is characterized by inflammation. The inflammatory process is partly initiated by bacterial translocation due to altered permeability [133] and is enhanced by mechanisms linked to microbiota metabolites, but also by the effect of bacteria and their components. Pathogenic and pathobiont microorganisms increased in obesity interact with TLR receptors, such as TLR-4 and TLR-5. These receptors recognize highly conserved microbial molecular motifs known as pathogen-associated molecular patterns (PAMPs). Among the bacterial components, lipopolysaccharide (LPS), a ligand of TLR-4, is an endotoxin present in the outer membrane of Gram-negative bacteria that induces inflammation [134]. Thus, as a result of interactions with TLRs, the transcription of NF-κB and proinflammatory cytokines is induced, contributing to the low-grade inflammation that characterizes obesity [135]. In this context, it has been shown that *L. rhamnosus* HDB1258 is able to modulate the immune system response in the presence or absence of LPS-induced inflammation. Thus, in the healthy host, it contributes to improving the innate immune response, such as cytotoxicity and phagocytosis mechanisms. During inflammation, it promotes the expression of IL-10 and decreases TNF-α, regulates the gut immune system, and shapes the composition of the gut microbiota, which helps maintain homeostasis [136].

In recent years, evidence for the role of LGG as a modulator of gut microbiota dysbiosis and intestinal permeability has been added in several models. The administration of LGG as a pretreatment for 4 weeks, prior to the induction of sepsis by cecal puncture in mice, demonstrated a greater expression of junction proteins (such as occludin), which was similar to that observed in the control group without sepsis [137]. In addition, LGG improved gut microbiota diversity, which was associated with an increase in intestinal barrier-regulating bacteria like the *Akkermansia* genus, which was decreased in septic mice [137]. These findings are similar to those identified in a murine model of inflammation [138]. Their results highlight that *L. rhamnosus* is able to modulate goblet cells to maintain the mucus layer, restore changes in lymphocytes both locally and systemically, and improve the expression of genes associated with intestinal permeability and the inhibition of endogenous proteases. In mice with obesity and non-alcoholic fatty liver disease, LGG supplementation has also been shown to improve the concentration of tight junction proteins, including claudin-1 and occludin in the proximal intestine, thereby improving gut–barrier function [107]. Likewise, in a model of obese mice that were fed a high-fat and fructose diet, supplementation with *L. rhamnosus LS-8* had beneficial effects on weight, insulin resistance, and inflammatory status [10]. Among the mechanisms identified to explain these results was the modulation of the microbiota by decreasing LPS-producing bacteria, *Proteobacteria,* and *Bacteroides* genus and increasing SCFA-producing commensal bacteria. Consequently, elevated levels of SCFA in this model were associated with a reduced expression of proinflammatory genes, such as TNF-α, IL-1β, IL-6, and MCP-1, as well as genes involved in lipid metabolism, such as PPARγ, Srebp-1c, CD36, Fabp2, and FAS. These findings are consistent with the identified effects of *L. rhamnosus* LRa05 supplementation in mice, where treatment improved microbial diversity and richness, with a decrease in *Streptococcus* that correlated with the regulation of blood glucose levels and carbohydrate metabolism in the liver [139]. Similarly, benefits of LGG supplementation in male Balb/C mice on a high-fat diet include a reduction in the relative abundance of Proteobacteria and the *Firmicutes*/*Bacteroidetes* ratio, along with an improvement in the ratio of villus height to crypt depth in the ileum [9]. In a clinical trial with participants over 60 years of age, *L. rhamnosus* supplementation generated changes in *Escherichia-Shigella, Akkermansia, Bacteroides,* and *Butyricimonas*, with only slight changes in alpha and beta diversity. These changes were associated with benefits in body weight, visceral fat, and lipid profile [140]. Altogether, these effects show the potential protective effect of *L. rhamnosus* on the gut environment in the context of obesity.

Prebiotics can help correct dysbiosis by suppressing the proliferation of harmful bacteria and promoting the growth of healthy bacteria, such as *Lactobacillus* and its metabolites. A clinical trial showed supplementation with oligofructose-enriched inulin as a prebiotic in overweight and obese children selectively improved gut microbiota composition, increasing the abundance of *Bifidobacterium* and reducing *Bacteroides vulgatus*. In turn, it improves body weight z-score and percent body fat, demonstrating its potential benefit in this condition [141].

The synbiotic combination of different prebiotics (such as inulin, mannose, fructo-oligosaccharides, acanthopanax senticosus polysaccharide, chitosan oligosaccharide, saposhnikovia divaricata polysaccharide, polysaccharide of atractylodes macrocephala koidz, astragalus polysaccharides, Yu–Ping–Feng polysaccharide, and xylooligosaccharides) with *L. rhamnosus* has been evaluated in vitro [142,143]. This has shown that the combination with inulin favors the survival of LGG during storage of *L. rhamnosus*, while the combination with fructo-oligosaccharides provides the best effects in terms of the proliferation of the probiotic [144]. In addition, synergistic effects in the modulation of the microbiota, in the production of SCFA [143], as well as the activation of anti-hyperlipidemic mechanisms [142] from the isolated administration of prebiotic and probiotic, have also been identified. With in vivo studies, it has been proven that the administration of *L. rhamnosus* BST-L.601, together with mashed sweet potato paste as a source of prebiotic, was more effective than the administration of the isolated microorganism to prevent weight gain in mice fed a high-fat diet [23]. Among the mechanisms that have been identified, suppressing lipogenesis and adipogenesis stand out. These benefits can also be explained in part by the particular effect of the bioactive compounds present in sweet potato paste since it contains resistant starch, caffeic acid, phenolic compounds, anthocyanins, and caffeoyl compounds [145], which have separately demonstrated anti-obesogenic, lipid-lowering, and microbiota-modulating benefits [146]. Other synbiotic combinations have been tested, such as *Cudrania tricuspidata* extract with *L. rhamnosus* 4B15, which have shown an enhanced effect, as compared to their individual administration to obese mice. The combination modulated the abundance of *Clostridiaceae, Lactobacillus, Helicobacter,* and *Ruminococcus*, among others. In relation to functionality, various pathways were up-regulated, such as those related to carbohydrate, lipid, and amino acid metabolism [147], which provides evidence of the enhanced effect of *L. rhamnosus* when administered together with a prebiotic.

Regarding postbiotics, the protein-based LGG postbiotic (HM0539 hydrolase) [148] has been identified as capable of prophylactically reducing susceptibility to systemic neonatal infection with *E. coli* K1 in rats, its translocation and systemic dissemination, associated with beneficial effects on intestinal integrity, and immune modulation. Among the protective mechanisms in the intestinal barrier, it promotes mucin expression and prevents LPS-induced damage and TNF-α production. In addition, HM0539 hydrolase negatively regulates the expression of zonula occludens-1 (ZO-1), which, together, contributes to maintaining epithelial integrity and promoting the maturation of the gut-defense system [149].

The identification of p40 and p75 proteins isolated from culture supernatants of LGG clarified the nature of the molecule responsible for the inhibition of apoptosis in mouse colonic epithelial cells [150]. These proteins were also implicated in the regulation of proliferation and survival of intestinal epithelial cells [150]. This led to the recent development of the recombinant protein p40 (rt-p40) [151], obtained from *L. rhamnosus* GR-1, which was able to increase in vitro occludin expression in HaCaT keratinocyte epithelial cells.

Lipoteichoic acid from the supernatant of *L. rhamnosus* has shown anti-inflammatory and preventive potential against intestinal barrier damage, which could positively contribute to the prevention and modulation of obesity. This is because lipoteichoic acid isolated and purified from *L. plantarum* A3, *L. reuteri* DMSZ 8533, and *L. acidophilus* CICC 6074 has an anti-inflammatory effect associated with the decrease in pro-inflammatory cytokines and an increase in anti-inflammatory cytokines. This is achieved by blocking the activation of the MAPK and NF–κB pathways in LPS-induced RAW264.7 cells [152]. The presence of lipoteichoic acid in extracellular vesicles (EV) also plays a role in the interaction and binding of p40 and p75 proteins [153]. This could explain its effect in reducing inflammation and apoptosis of the intestinal epithelium and disrupting the integrity of the intestinal barrier [150,151], which in turn could contribute to its effect on obesity.

**Figure 2:**
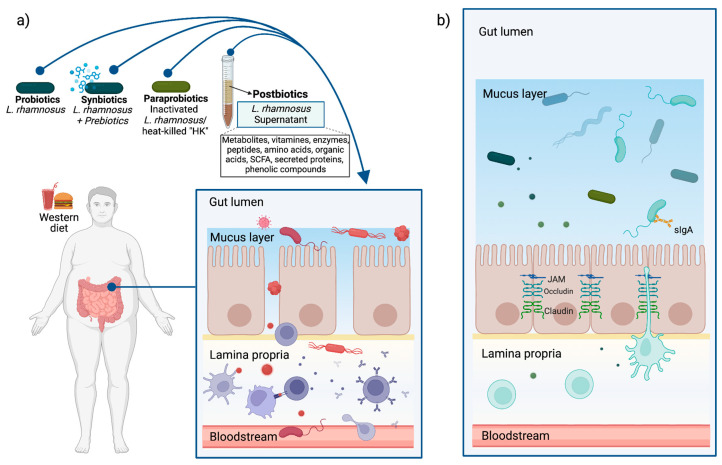
Possible beneficial effects of *L. rhamnosus* on obesity-associated leaky gut syndrome. (**a**) Obesity is associated with dysbiosis and increased intestinal permeability, which causes the translocation of bacteria, toxins, and dietary antigens, which stimulate the mucosal immune system and trigger a proinflammatory response. The use of live *L. rhamnosus* (probiotic), heat-killed (paraprobiotic), and/or its components or products (postbiotics) could contribute to the restoration of epithelial integrity by (**b**) improving the state of dysbiosis, modulating mucus production, and inducing the expression of tight junction proteins, preventing bacterial and antigen translocation and, consequently, the activation of the immune response. Custom image created with BioRender.

The aforementioned evidence suggests that the protective effect on the intestinal barrier could also be promoted by the components and metabolites of *L. rhamnosus,* and not only by the living microorganism. It also highlights the importance of identifying the compounds responsible for the beneficial effects, which could facilitate the development of therapeutic methods for leaky gut associated with chronic diseases, including obesity. Figure 2 summarizes the described effects regarding the protection of the intestinal barrier.

## 7. Hunger–Satiety Pathway

Obesity is undoubtedly associated with hyperphagia and the hunger–satiety pathway. The hypothalamus in the central nervous system integrates central and peripheral signals that regulate metabolism and hunger–satiety responses. This structure contains the arcuate nucleus, which has two populations of neurons: neuropeptide Y (NPY) neurons, which, through the co-secretion of NPY peptides and agouti-related protein (AgRP), exert an orexigenic effect. In contrast, proopiomelanocortin (POMC) neurons, through alpha-melanocyte-stimulating hormone (α-MSH) and cocaine- and amphetamine-regulated transcript (CART), exert an anorexigenic effect that reduces food intake [154].

Peripheral tissues, mainly the gastrointestinal tract and adipose tissue, secrete hormones involved in this pathway. Hormones derived from the gastrointestinal tract include ghrelin [155], cholecystokinin (CCK) [156], glucagon-like peptide-1 (GLP-1) [157], PYY, and insulin. Adipose tissue, mainly through leptin, provides an anorexigenic effect at the central level. GLP-1, leptin, and insulin activate POMC at the central level, resulting in an anorexigenic effect. In contrast, ghrelin activates NPY/AgRP neurons and is inactivated by insulin, CCK, PYY, and leptin [158].

GLP-1 is an incretin-type peptide synthesized by L-type cells of the ileum and proximal colon that, when released, stimulates its receptor in afferent vagal neurons and neighboring tissues like the pancreas, resulting in a central anorexigenic effect, a decrease in gastric emptying, and stimulation of glucose-dependent insulin release from β-pancreatic cells [159]. The presence of the enzyme dipeptidyl peptidase IV (DPP-IV) limits the half life of GLP-1 since this enzyme promotes its inactivation, which is the mechanism of action of new antidiabetic drugs capable of inhibiting this enzyme and, thus, promoting a higher concentration of GLP-1 [160].

Enterohormones respond to the presence of nutrients in the intestine, as well as to metabolites derived from the digestion of the gut microbiota [161], so substances produced by the gut microbiota may have an impact on areas in the central nervous system that regulate hunger and satiety via the gut–brain axis.

Some metabolites produced by *Lactobacillus* spp. have been shown to be responsible for an effect on the regulation of hunger/satiety. These include SCFAs like acetate, propionate, and butyrate, which have been shown to be among the main metabolites produced in fermentation processes [162]. *L. rhamnosus* as a probiotic produces SCFAs, including acetic, propionic, and butyric acids from different types of polysaccharides, such as those derived from edible mushrooms [163] and pistachio [164]. However, when the probiotic is cultured in a conventional medium, the production of SCFAs can be modified. Regarding *L. rhamnosus* SD4, *L. rhamnosus* SD11, and LGG, they produce butyrate [165], while *L. rhamnosus* ATCC 53103 produces acetic acid, although butyric and propionic acid were not detected [63].

SCFAs have been associated with the modulation of enterohormones. The mechanisms by which SCFAs could stimulate the release of these hormones have been described [166], which include a mechanism mediated by the free fatty acid receptors type 2 and 3 (FFAR2 and FFAR3, also known as GPR43 and GPR41, respectively) [167] and the olfactory receptor 51E2 [65,166]. In particular, the binding of propionate to FFAR2 suggests that it regulates the synthesis and release of GLP-1, while its binding to FFAR3 regulates GLP-1, PYY, and, potentially, leptin [167]. Additionally, SCFAs have been associated with a decrease in circulating ghrelin, through a mechanism of action at the FFAR2 receptor, as well as antagonism at the ghrelin receptor GHR1a [168,169]. SCFAs have also been shown to stimulate leptin expression through FFAR3 on adipose tissue [170], as well as modulate vagal nerve signaling that communicates with the hypothalamus for central appetite regulation [171,172,173]. However, the precise mechanistic pathways involved in the release of these enterohormones by SCFAs [166], especially those derived from supernatants, remain to be elucidated.

Among the anti-obesogenic effects associated with the administration of SCFA, a study by Jiao et al. [64] compared the effect of the administration of 3 SCFA (acetate, propionate, and butyrate at 5%) for 35 days in the regulation of appetite in vivo (C57BL/6J mice). They observed that acetate increased serum GLP-1 and leptin (*p* < 0.05), while propionate increased serum GLP-1, PYY, and leptin (*p* < 0.05). In the case of butyrate, it increased serum PYY (*p* < 0.05) and was the only one to decrease daily food intake (*p* < 0.05). Regarding changes in gene expression, propionate and acetate increased leptin mRNA in epididymal adipose tissue, which coincided with its serum concentration (*p* < 0.05). In contrast, butyrate partially prevented this increase. No differences in adiponectin mRNA were observed. These results indicate the potential modulating effect of SCFAs on the hunger–satiety pathway by regulating GLP-1, PYY, and leptin.

Butyrate increased plasma concentration of peripheral hormones (GLP-1 and PYY *p* < 0.05; GIP and insulin *p* < 0.001), while propionate increased GIP (*p* < 0.001) and insulin (*p* < 0.05) in the C57BL/6N mice model (10 min; 400 mg/kg body weight) [65]. The same study also showed that supplementing a high-fat diet (HFD) with SCFAs over a 4-week period was associated with a complete prevention of weight gain in response to butyrate (5% *w*/*w*) and propionate (4.3%) (*p* < 0.0001). This was also associated with a decrease in cumulative food intake (butyrate *p* < 0.05, propionate trend not significant). Acetate showed no changes in these hormones [65].

Propionate, in particular, has been shown to stimulate the secretion of GLP-1 and PYY in an in vivo pig model, administered through a cecal fistula over a 28-day-period. In this study, propionate infusion decreased food intake in the short term (0–2 h; *p* < 0.05), but not in the medium term (4 weeks), and stimulated the colonic release of PPY and GLP-1 (*p* < 0.05). Plasma PYY increased at the end of the experimental period (*p* < 0.05). In this study, propionate also showed an influence at the central level by regulating the downstream expression of AgRP mRNA (*p* < 0.05) and CART mRNA upstream (*p* = 0.09), both in the hypothalamus [174]. Additionally, propionate has shown significant stimulation for the release of anorexigenic hormones PYY and GLP-1 in an in vitro colonic cell model [175]. Later in this investigation, a randomized double-blind placebo-controlled clinical trial was conducted in 49 subjects. After 24 weeks of dietary supplementation with 10 g of inulin/propionate per day, it was shown that it reduced energy intake (*p* < 0.01) and had a greater weight loss at the end of this period, although this effect was not significant between groups. No significant postprandial difference in plasma PYY or GLP-1 levels was observed with long-term supplementation (24 weeks). However, a postprandial increase in these hormones was observed, mostly between 240 and 420 min after oral supplementation.

LGG postbiotics have shown another mechanism by which they could exert satiety-modulating effects. The DPP-IV inhibitory capacity (%) of a postbiotic (supernatant) and postbiotic obtained by ultrasonic treatment of different *Lactobacillus* strains showed that the postbiotic obtained by ultrasonic from LGG has DPP-IV inhibitory capacity (6.2 ± 1.4), while the supernatant showed none [92]. In contrast, supernatants from *L. acidophilus* KLDS1.1003 and *L. bulgaricus* KLDS1.0207 showed inhibitory capacity (7.1 ± 0.3 and 5.5 ± 0.3, respectively). Although the ability of *L. rhamnosus* species and the LGG strain to increase serum GLP-1 levels in vivo as probiotics has been confirmed [92], the effect of postbiotics on serum GLP-1 levels associated with an anorexigenic effect requires further study.

**Figure 3:**
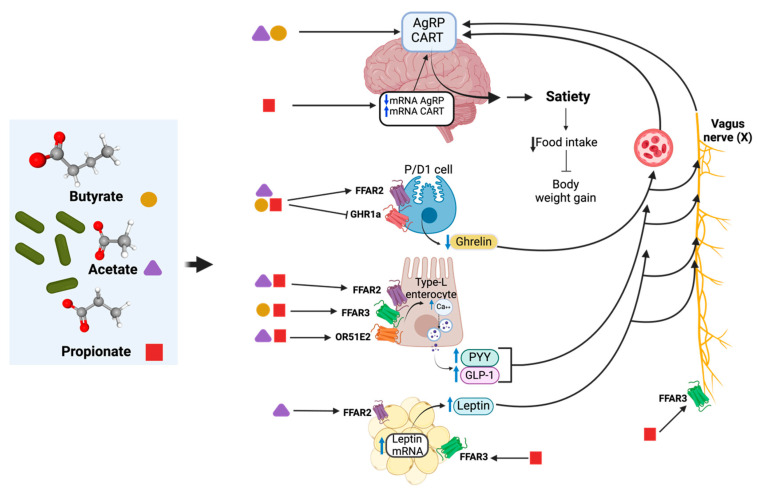
At the central level, SCFAs modulate the regulatory centers of AgRP and CART to induce satiety, which is partly associated with changes to their mRNA expression. Peripherally, in the gastrointestinal tract, SCFA decreases the release of ghrelin through the FFAR2 and GHR1a receptors in gastric P/D1 cells. On the other hand, in L-type enterocytes, SCFA may regulate signaling pathways that induce the synthesis and secretion of GLP-1 and PYY through the FFAR2, FFAR3, and OR51E1/2 receptors. In adipose tissue, SCFAs induce leptin gene expression, which is associated with its circulating concentration. SCFAs centrally regulate appetite through the FFAR3 receptor and activation of vagal nerve signaling. Enterohormones are synthesized as a result of SCFAs upon entering the systemic circulation and activating their corresponding receptors in afferent nerve pathways, together regulating hunger/satiety centers in the hypothalamus, thereby inducing satiety and preventing body weight gain. Custom image created with BioRender. SCFA structures taken from PubChem.

Although most of the studies discussed did not use SCFAs from *L. rhamnosus*, it is capable of producing them under different culture conditions. Thus, it is possible to suggest that the anti-obesogenic potential of *L. rhamnosus* is due in part to SCFAs, which partially modulate different mediators of the hunger/satiety pathway. Figure 3 schematizes the effects of SCFAs potentially produced by *L. rhamnosus* in modulating the hunger/satiety pathway directly and indirectly at the peripheral and central levels. These findings would add to the other beneficial effects observed from these compounds, such as anti-inflammatory, antimicrobial, and immunomodulatory effects, promoting intestinal integrity and adequate permeability, among others [176]. However, additional research is required to clarify the mechanisms by which SCFA and other postbiotics from *L. rhamnosus* could modulate hunger/satiety at the peripheral and central levels, as well as examine their short-, medium-, and long-term effects.

## 8. Perspectives

Despite the limited number of studies on the anti-obesogenic effect of postbiotics and paraprobiotics, in particular, compounds or molecules responsible for exerting modulatory effects on obesity, it is possible to distinguish some common factors among the available studies. Among them:

(1) The energy source and nutrients used for bacterial growth and supernatant production are the cause of different results between studies [148]. This considers the properties of the culture, such as whether it is a standardized culture medium, a food, or a combination of both. Even though there is ample variability and availability of standardized culture media (e.g., MRS), the products used in fermentation and the production of postbiotics (e.g., supernatants), are generally of plant origin [177] that are rich in bioactive compounds like anthocyanins, polyphenols, flavonoids, anthocyanins, organic acids, and amino acids, in addition to macronutrients like carbohydrates, proteins, and lipids, as well as micronutrients like vitamins and minerals [178]. The process of bacterial food fermentation can modify its nutrient and metabolite profile [179], resulting in changes in the total phenolic content [59,178]. This could indicate an increase in the bioactivity and bioaccessibility of these compounds [180], as demonstrated in supernatants derived from ferments with a high content of compounds that may positively influence the benefits observed in obesity [181].
  Further studies are needed to evaluate the supplementation of a standardized culture medium with food of plant origin in order to potentially improve the biological activities of the probiotic, paraprobiotic, and postbiotic forms [182]. This could allow the development of new standardized media and suitable experimental models for specific paraprobiotic and postbiotic therapeutic research. However, the fermentation process is not always accompanied by an increase in total phenolic content or other compounds, which could influence the expected beneficial effects [183].
(2) Environmental factors: Other than nutrient content, identifying other culture conditions that can enhance the concentration of metabolites or postbiotic components of interest. In this regard, pH and inoculum size significantly affected exopolysaccharide (EPS) production from *L. plantarum* R301 (*p* < 0.05), as well as MgSO_4_ concentration (*p* = 0.2576). Furthermore, glucose was found to be the most favorable source for EPS production by *L. plantarum* R301. After providing the appropriate media, the strain increased EPS production by 84.70% [184].
  Additionally, the evaluation of butyrate production capacity by different *Lactobacillus* strains revealed that the production of this SCFA was highest in different time frames for different strains (24 vs. 48 h) and increased with temperature, with the highest production observed at 45 °C for all strains [165]. In addition to the limitations of the used culture medium (MRS) and the available nutrients for SCFA synthesis in the aforementioned study, these findings highlight the importance of considering temperature and incubation time to optimize the production of a product or metabolite.
  Taken together, these results show the importance of identifying and applying appropriate environmental factors and conditions that promote the production of the postbiotic or metabolite of interest by the microorganism, which will facilitate the presence and study of these compounds.
(3) The production of beneficial postbiotics can be positively influenced by other types of bacterial species, which supports taking into consideration the use of multiple bacterial strains. In support of this, a recent study by Song et al. [185] evaluated the benefits of different postbiotic and paraprobiotic forms, administered together or alone, in an in vivo intervention rat model. Oat-derived postbiotics were obtained from *L. plantarum* HH-LP56, *L. reuteri* PB-LR09, *L. rhamnosus* PB-LR76, *L. acidophilus* HH-LA26, and *B. lactis* HH-BA68, used together as a treatment group (OF-5) or separately in another group (OF-1; *L. plantarum* HH-LP56). Paraprobiotic forms were HK and employed together as a treatment group (HK-5). All forms resulted in a complete restoration of serum glutathione peroxidase (GSH-PX) levels. However, HK-5 and OF-5 were superior in restoring serum SOD levels, and only OF-5 prevented elevation of MDA levels. Regarding hepatic inflammation, all forms were able to reduce hepatic TNF-α protein expression in a similar manner. Moreover, lipid metabolism markers showed a superior effect of the postbiotics (OF-1 and OF-5) by regulating the protein expression of PPAR-α, FAT/CD36, and FaβO, which was not shown by Bac-5. OF-5 was superior in other parameters such as NAFLD activity score (NAS) and liver/body weight ratio. The above demonstrates that different postbiotic and paraprobiotic forms resulted in different effects. The administration of oat-based postbiotics derived from five different probiotics, which resulted in altogether superior protective effects in terms of lipid and hepatic changes through regulation of protein expression (translational level) when compared to the use of only one or the HK form [185]. This can be attributed to a synergistic effect between the selected probiotics, as well as the composition of metabolites generated in the ferment, which invites the evaluation of the benefit of using selected strains together versus separately.
  Additionally, gut microbiota composition may influence the outcomes. Lützhøft et al. [186] used an in vivo model with Göttingen minipigs and found that 12 weeks of exposure to an HFD resulted in a decrease in intestinal microbiota species richness, which was associated with a decrease in circulating SCFAs and other metabolites like hippuric acid, xanthine, and trigonelline, as well as an increase in branched amino acids. Similarly, a study by Krolenko et al. [187] confirmed the decrease in SCFA in an in vivo model of diet-induced obesity and compared it with genetically induced obesity. They concluded that diet has a greater influence on the production of these metabolites.
  Taken together, these results suggest that other bacterial strains may influence interbacterial interactions that may also influence the production of desired metabolites for the postbiotic form. This should also be considered to ensure that postbiotics used for therapeutic purposes in the future are associated with changes in the diet and other factors that positively modulate the gut microbiota.
(4) Postbiotics (e.g., supernatants) can modulate metabolism at a transcriptional and translational level by regulating mRNA expression and protein expression, respectively. This results in the modulation of a variety of signaling pathways. Other reported mechanisms include the modulation of enzymatic activity and the immune response (this is discussed extensively by Jastrząb et al.) [188].
(5) Microorganisms can be used as “machinery” for the production of postbiotics, but, if included, it is necessary to inactivate them [12]. Some cellular fragments are necessary to observe the beneficial effects, such as lipopolysaccharides, intracellular proteins, S-layer proteins, and lipoteichoic acid, among others [188]. Continuing research with the paraprobiotic form will enable the identification of the components that exert specific beneficial effects on obesity, thereby offering potential treatment options.
(6) Implications for clinical development: Probiotics have long been used, and clinical trial studies are extensive. Postbiotics and paraprobiotic forms, on the other hand, are newly researched areas with unidentified gaps. They are promising therapeutic agents in the prevention and intervention of obesity since their positive effects not only regulate gene expression in target organs and tissues implicated in obesity’s alterations, such as adipose tissue (lipid deposition and hormonal regulation), the brain (hunger–satiety and gut–brain axis) and the modulation of intestinal microbiota for added effects. So, what is stopping postbiotic and paraprobiotic *Lactobacillus* clinical application?

In support, some postbiotics show beneficial effects only when exposed to pathological environments or stress conditions, such as obesity. This may be because some microorganisms synthesize certain components or enzymes to protect themselves from stress situations [184]. Even though this could put into question their preventive use, preventive positive effects in preventive animal models of obesity show the beneficial effects are maintained [23,24,71].

Regarding application, postbiotic and paraprobiotic could initiate their action as soon as administrated, which could provide a faster start of action without considering an appropriate “colonization” [189]. Additionally, postbiotic and paraprobiotic forms result in easier handling conditions and requirements when compared to their living forms. Regarding safety, the potential antibiotic resistance development or opportunistic infection by postbiotic and paraprobiotic forms could be disregarded, which, in a leaky gut setting and when the changes in immune function are present in obesity, is advantageous. These forms may lack pathogenicity and have a reduced risk of bacteremia, which should also be considered in vulnerable populations, such as immunosuppressed, children, or older subjects. This could allow its application in an attempt to approach the high child and adult obesity trends [190]. However, as with any therapeutic agent, it is essential to investigate potential adverse effects [191].

In order to further explore safety in humans, evaluation of pharmacokinetic parameters is required. This will also determine therapeutic doses, including quantity and frequency [192], to finally address the challenge of standardized doses, which will allow progress in this field.

Postbiotic and paraprobiotic forms accomplish their beneficial effects when administered orally and exposed to gastrointestinal tract conditions, which is a desired property for a therapeutic agent. Notwithstanding, some studies have explored strategies to enhance postbiotic delivery to targeted sites in combination with prebiotics for a synergistic result upon interfacing with the intestinal microbiota [193]. Alternatively, there is a continuous evaluation of incorporating these combinations into food products to develop fermented and/or functional foods [194].

Some clinical trials have demonstrated the efficacy of postbiotics and paraprobiotics in human disorders and conditions. Some fields include dermatology: postbiotics in acne [193] and burn wounds in the phase 1 trial [195]; allergology: paraprobiotics (heat-killed) *Bifidobacterium* and *Streptococcus* on infant formula decreased allergic adverse events in children with family history of atopy during the first 2 years of life (n = 129, experimental n = 66) [196]; and paraprobiotic (bacterial lysate) OM-85 Broncho-Vaxom (BV) (complex of oral bacteria) in children with asthma (n = 60, experimental n = 24), decreased asthma attacks associated with positive modulation of peripheral blood NKT cells, and CD4^+^ NKT reduction of IL-4 [197]. The sum of other studies examining the immune-regulating effect of postbiotic and paraprobiotic in children have yielded multiple results, yet these forms have been regarded as safe and recommended for use [198].

One paraprobiotic (pasteurized inactivated bacteria *Akkermansia mucinphila*) has been proven in a randomized, double-blind, placebo-controlled pilot study in overweight and obese subjects (n = 32) to improve insulin sensitivity and dyslipidemia and, to a lesser extent, decrease body weight and fat mass [199].

In summary, the use of *L. rhamnosus* as postbiotics or paraprobiotics in both children and adults predisposed to obesity or in the obese state is subject to opportunity and holds significant promise. This will unequivocally support advancements in clinical applications.

## 9. Conclusions

*L. rhamnosus* in its probiotic, postbiotic, and paraprobiotic forms appears to exert a modulating effect on different obesity-associated factors, ranging from the regulation of the hunger/satiety pathway, adipogenesis, thermogenesis, inflammation, oxidative stress, and the restoration of altered intestinal permeability, as evidenced by changes of particular biomarkers of each pathway through a regulatory capacity at the transcriptional and translational level. The beneficial effects of *L. rhamnosus* and its postbiotics and paraprobiotics on obesity could be improved by interbacterial interactions with selected strains, as well as the conditions and available nutrients of the medium. Information regarding the compounds responsible for the benefits observed in obesity is limited. Therefore, it is necessary to continue the research to protocolize the findings for their reproducibility and application and identify, as much as possible, the substances present and responsible for the beneficial effects in order to provide progress in this field. This will lead to a better understanding of the mechanisms of action of metabolites and/or products derived from bacteria and, thus, expedite their clinical and therapeutic application.

## Figures and Tables

**Table 1:** Summary of *Lactobacillus rhamnosus*-based postbiotic anti-obesity treatments, according to the main compounds responsible for the observed effects.

Medium Conditions and/or Extraction	Compound	Model	Main Obesity-Related Results
Cell-free Supernatant of *L. rhamnosus* ATCC 7469 incubated in liquid MRS medium [34]	Inosine	In vitro; BAT explants WT mice (C57BL/6). Inosine (100 µg/kg)	Stimulates energy expenditure in brown adipocytes through the cAMP–protein kinase A-signaling pathway (cAMP-PKA) [35]
	O-succinyl-L-homoserine	Humans; 19 premature infant serum samples. GC-MS analysis.	Associated with low serum levels of O-succinyl-L-homoserine [36]. O-succinyl-L-homoserine is a precursor to methionine. Methionine restriction decreases body weight and adiposity in vivo [37].
Supernatant of brewed coffee extract, fermented with LGG, analyzed by LC-QTOF-MS/MS [33]	Pyroglutamic acid	In vitro; *L. acidophilus* and *Bifidobacteria* in yogurt for 30 days. Determined by HPLC.	Potential antioxidant capacity [38]
		Humans; 103 middle-aged abdominally obese men and women in 4 groups of different exercise schemes with 6 months duration. LC-MS/MS plasma samples.	Potential biomarker of cardiometabolic health: inverse correlation with visceral adipose tissue deposition in humans [39]
		In vivo; Goto–Kakizaki diabetic rats (n = 5). Diet containing 0.05% pyroglutamic acid for 43 days.	Decreased epididymal adipose tissue, improvement in lipid profile, and decreased serum insulin and TNF-α levels, as well as hepatic total cholesterol and triglycerides (*p* < 0.05) [40]
	Indole-3-lactic acid (ILA)	In vitro; human adipose-derived mesenchymal stem cells (hMSCs). Various concentrations.	Antiadipogenic concentration-dependent, evidenced by Oil Red O-staining microscopy and quantification of lipid droplet accumulation [41]
		In vitro; human intestinal microbiota, ILA (172 mg/L), 24-h fermentation.	Potential to regulate the intestinal microbiota by increasing the average relative abundance of *Firmicutes* and *Bacteroidota phyla* (9.27% and 15.38%, respectively) and decreasing *Proteobacteria* (14.36%). Increase in acetic acid, propionic acid, and butyric acid [42].
		In vivo; DSS-induced colitis in C57BL/6 mice. ILA (20 mg/kg, oral) for 7 days.	Anti-inflammatory (serum TNF-α, IL-1β, *p* < 0.05 vs. DSS) and gut microbiota regulator [43]
	Indole-3-carboxaldehyde	In vivo; C57BL/6 mice, DSS-induced ulcerative colitis. Indole-3-carboxaldehyde by gavage for 7 days.	Modulation of intestinal integrity. Anti-inflammatory by inhibiting intestinal TLR4/NF-κB/p38 pathway [44]
	3-Phenyllactic acid	In vivo; C57BL/6 mice fed an HFD (60% fat) for 5 weeks. 3-Phenyllactic acid supplemented in drinking water.	3-Phenyllactic acid regulates lipid metabolism by activating intestinal PPAR-γ, which inhibits lipid secretion in intestinal epithelial cells and prevents excessive adiposity [45]
		In vitro; differentiated hMSCs.	Antiadipogenic by reducing lipid droplet accumulation evidenced by Oil Red O staining [41]
	Hydroxydodecanoic acid isomer	In vitro; 3T3-L1 cells.	(3R)-3-hydroxydodecanoic acid from *Bauhinia divaricata* L. extract may exert anti-obesogenic effects by inhibiting adipocyte differentiation [46]
	Quinic acid	In vivo; intervention model on Swiss Albino mice fed an HFD (58% fat) for 8 weeks, followed by oral administration of quinic acid (75, 150, 300 mg/kg) for 6 weeks.	Prevents steatosis, dyslipidemia, and weight gain [47]
		In vivo; C57BL/6J ApoE^−/−^ mice fed an HFD. Quinic acid (10 mg/kg, intraperitoneal) 5 times a week for 12 weeks.	Regulates gut microbiota; inhibits atherogenesis [48]
	5-Hydroxyferulic acid	In vitro; 3T3L1 preadipocytes. 5-Hydroxyferullic acid isolated from wasabi leaves.	Inhibition of adipocyte differentiation and lipid accumulation by suppressing gene expression of PPARγ and C/EBPα and genes involved in adipogenesis like SREBP-1c, ACC, and FAS [49]
	4-Vinylphenol	In vivo; C57BL/6J mice fed an HFD (45 kcal% fat) supplemented with rice husk extract (*O. sativa* L.) (20 or 40 mg/kg) by oral gavage daily for 14 weeks.	4-vinylphenol identified as one of the most abundant compounds. The extract reduced adipose tissue and liver adipogenesis and improved lipid profile [50]
LGG in MRS broth, for 48 h under anaerobic conditions. Collection of supernatant and metabolite analysis by HPLC-UV/DAD [51]	p-Hydroxyphenyllactic acid [33]	In vitro; secreted by *L. fermentum* CECT5716.	Antioxidant capacity [52]
		In vitro; hepatic mitochondria and blood neutrophils from Wistar rats.	Decreased ROS production in neutrophils and mitochondria [53]
	Ferulic acid (FA)	In vivo; Wistar rats fed a supplemented HFD with FA (2 g/kg) for 8 weeks.	Increased plasma antioxidant capacity, reduced body weight, adipose tissue, dyslipidemia, and inflammation [54]
Lysed cells of *L. rhamnosus* increased the production of lactoyl-phenylalanine compared to its viable form, exposed to amino acids as precursors. Compositional analysis by UPLC/ESI-MS. [55]	N-Lactoyl-phenylalanine [33]	In vivo; mice and racehorses for level determination [56] In vivo; diet-induced obese mice, N-lactoylphenylalanine (50 mg/kg, intraperitoneal) [56] Humans; sedentary overweight and obese subjects. Determination of N-Lactoylphenylalanine before and after 8-week exercise intervention [57].	Levels increase immediately after running. Acute administration of N-lactoylphenylalanine suppressed food intake by ~50% in obese mice but not lean mice [56] Appetite suppression, decreased adiposity and decreased body weight [56,57].
Theobroma grandiflorum juice ferment by *L. rhamnosus* ATCC9595. Analyzed by LC-QQToF [58]	Vanillic acid (VA)	In vivo; melanoma model on C57BL/6J mice fed HFD (60% fat) for 5 weeks. Afterwards, VA administered by oral gavage every other day for 2 weeks [59]. In vivo; C57BL/6J mice with diet-induced obesity, HFHFD (25% fructose, 25% lard), and VA (0.5% *w*/*w*) diet supplementation [60].	Decreased adiposity. Promotes thermogenesis and mitochondrial synthesis in brown and white adipose tissue through AMPK activation [59,60].
Supernatants, derived from the presence of pomegranate extract in SDM (semi-defined media) with LGG [61]	Glutamine	Ex vivo; subcutaneous abdominal WAT of obese subjects (n = 52). In vivo; male C57BL/6 mice fed an HFD for 5 weeks, intraperitoneal glutamine for the last 2 weeks.	Low levels identified in WAT from obese individuals and inversely associated with body fat mass percent; probably related to reduced glutamine synthetase in the obese state. Attenuates the expression of proinflammatory genes (IL-6, IL1-b, CD68) in adipocytes and macrophage infiltration in eWAT [62]
Supernatant of *L. rhamnosus* ATCC 53103 derived from BHIG (brain heart infusion glucose culture) culture inoculum. Analyzed by HPLC-MS-MS [63]	Short-chain fatty acids: Acetate	In vivo; C57BL/6J mice fed an HFD with 5% sodium acetate for 35 days.	Acetate increased serum GLP-1 and leptin (*p* < 0.05), associated with increased relative expression of leptin mRNA in epididymal fat (*p* < 0.05) [64]
	Propionate	In vivo; C57BL/6J mice fed an HFD with 5% sodium propionate for 35 days.	Propionate increased serum GLP-1, PYY, and leptin (*p* < 0.05), associated with an increase in leptin mRNA (*p* < 0.05) [64]
	Butyrate	In vivo; C57BL/6J mice fed an HFD with 5% sodium butyrate for 35 days.	Butyrate increased serum PYY (*p* < 0.05) and decreased daily food intake (*p* < 0.05) [64]
		In vivo; C57BL/6N mice fed an HFD (60% kcal from fat).	Butyrate exerts short-, medium-, and long-term beneficial effects: – Acute (10 min) administration of sodium butyrate (400 mg/kg body weight) increased plasma GLP-1, GIP, PYY, and insulin (*p* < 0.05). – Administration of butyrate (5%) for 9 days together with a HFD decreased cumulative food intake (*p* < 0.05). – Administration of butyrate (5%) for 4 weeks together with a HFD prevented weight gain (*p* < 0.0001) [65]

MRS (Man-Rogosa-Sharpe); cAMP-PKA (Cyclic Adenosine 3′,5′-Monophosphate Protein Kinase A); LC-QTOF-MS/MS (Liquid Chromatography-Quadrupole Time-of-Flight Tandem Mass Spectrometry); TNF-α (Tumor Necrosis Factor Alpha); TLR4/NF-κB/P38 (Toll-Like Receptor 4/Nuclear Factor-κB/P38); PPAR-γ (Peroxisome Proliferator-Activated Receptor Gamma); ApoE^−/−^ (Atherosclerosis-Prone Apolipoprotein E-Deficient); C/EBPα (CCAAT/Enhancer Binding Protein A); SREBP-1c (Sterol Regulatory Element Binding Protein 1c); ACC (Acetyl-Coa Carboxylase); FAS (Fatty Acid Synthase); HFD (High-Fat Diet); hMSCs (human adipose-derived mesenchymal stem cells); HPLC-UV/DAD (Optimized Liquid Chromatography With Diode Array Ultraviolet Detection); ROS (Reactive Oxygen Species); UPLC/ESI-MS (Ultra-Performance Liquid Chromatography-Electrospray Ionization Mass Spectrometry); LC-QTOF (Liquid Chromatography-Hybrid Quadrupole Time-Of-Flight Mass Spectrometry); LPS (Lipopolysaccharides); AMPK (AMP-Activated Protein Kinase); SDM (Semi-Defined Media); WAT (white adipose tissue); eWAT (epididymal white adipose tissue); I.P. (Intraperitoneal); IL-6 (Interleukin-6); IL-1B (Interleukin-1B); CD68 (Cluster Of Differentiation 68); BHIG (Brain Heart Infusion Glucose Culture); HPLC-MS-MS (High-Performance Liquid Chromatography With Tandem Mass Spectrometry); GLP-1 (Glucagon-Like Peptide-1); mRNA (Messenger RNA); PYY (Peptide YY); Agrp (Agouti-Related Protein); CART (Cocaine- And Amphetamine-Regulated Transcript); GIP (Gastric Inhibitory Polypeptide).

## Data Availability

No new data were created or analyzed in this study. Data sharing is not applicable to this article.
